# Bifocal Stabilisation of Acute Acromioclavicular Joint Dislocation using Suture Anchor and Temporary K-Wires: A Retrospective Analysis

**DOI:** 10.5704/MOJ.2211.016

**Published:** 2022-11

**Authors:** S Vijayan, MS Kulkarni, CP Jain, S Shetty, MN Aroor, SK Rao

**Affiliations:** Department of Orthopaedics, Kasturba Medical College Manipal - MAHE, Manipal, India

**Keywords:** acromioclavicular joint, coracoclavicular joint, joint dislocations, open reduction, shoulder injuries

## Abstract

**Introduction:**

The acromioclavicular joint (ACJ) is a major link connecting the upper limb to the torso. The acromioclavicular and coracoclavicular (CC) ligaments help in stabilising the joint. We feel it is prudent to address both these ligament injuries, to achieve optimum result. This study was undertaken to analyse the results of a simple frugal surgical technique we used to deal with this injury considering stabilisation for both these ligaments.

**Materials and methods:**

In this retrospective study, skeletally mature patients with Type III, IV or V ACJ dislocations who underwent open reduction and stabilisation of the joint with temporary K-wires, repair of the capsule and augmentation of CC ligaments with suture anchors were included. Clinico-radiological and functional outcome was evaluated. Functional assessment of the upper limb was analysed using the Disabilities of Arm, Shoulder, and Hand Score (DASH), Constant shoulder score (CSS) and Oxford shoulder score (OSS).

**Results:**

Clinical and radiological evaluation of the 32 patients who had completed two years from the index surgery, was done. Out of the 37 patients included initially, five were lost in follow-up. Majority of the subjects included were males and type V was the most common injury. Mean pre-operative CC distance on the affected side was 13.92±4.94mm. In the immediate post-operative radiograph, it was 7.63±2.08mm and in the final follow- up was 9.36±2.75mm. Measurements were taken by two independent investigators and inter, and intra-observer reliability were analysed by Interclass correlation coefficient. Excellent functional outcome was noted despite the 1.81±1.50mm average loss of correction. At final follow-up, mean DASH score was 4.67±4.18, Oxford shoulder score was 44.06±2.44 and Constant shoulder score was 86.37±5.81. The severity of the injury had no significant effect on the functional outcome post our method of stabilisation and rehabilitation.

**Conclusion:**

Bifocal fixation restores the multidirectional stability of the disrupted ACJ. Adequate radiological reduction, good functional outcome and simplicity of execution make this technique an undemanding one for use in regular practice.

## Introduction

Acromioclavicular joint (ACJ) forms one among the two articulating points between upper limb and the torso; another dynamic point of articulation being shoulder girdle and its muscular attachments. Direct injury due to fall on the tip of the shoulder or indirect injury due to fall on an outstretched hand can lead to disruption of the ACJ^1^. The severity of this injury increases as the successive structures fail, leading to dislocation of ACJ which is the basis for the most often used, Rockwood classification^2^.

Rockwood Type I and II injuries are treated successfully by conservative methods^3^. Controversy still exists about the management of Type III injuries^4^. However, in young and active patients with Rockwood type III to VI injuries, surgical intervention is preferred to get an anatomical reduction and good functional outcome^5^. There are more than 160 procedures which are described to treat these injuries^2,5^. These techniques can be broadly categorised into 4 types^5^. ACJ stabilisation techniques (K-wiring, hook plate, Tension Band Wiring)^6,7^, Coracoclavicular (CC) stabilisation techniques (CC ligament repair, augmentation with sutures, adjustable loop with button, Bosworth screw)^7-9^, excision of the lateral end of the clavicle with or without ligament reconstruction techniques and transfer of conjoint tendon or coracoacromial ligament to the lateral end of the clavicle (Weaver Dunn procedure)^6,10^. Of these, the last two types are used most commonly in chronic injuries, usually if they are more than three weeks old^6^.

Capsuloligamentous complex of AC joint particularly the superior and posterior acromioclavicular ligaments provide antero-posterior stability^10^. The coracoclavicular (CC) ligaments mainly prevent superior displacement. Hence it is prudent to address both these injured ligaments to get the optimum results^11^. There are not many studies in the literature which describe the outcome after stabilisation of both ACJ and CC disruption^12-14^. Certain techniques do not deal with stabilisation of both ligaments while some have a longer learning curve. In this study, we describe an easily executable and frugal surgical technique for managing these injuries by stabilising both these ligaments and further clinico-radiologically and functionally evaluated them for a period of two years.

## Materials and Methods

A retrospective study was conducted on patients operated with ACJ dislocation at our centre by the said technique between 2012 and 2018. Informed written consent from patients and Institutional ethical committee clearance for this retrospective study was obtained. Our study meets the international ethical standards as per the Helsinki declaration^15^.

Skeletally mature patients who were treated by open reduction, transfixation of the dislocated ACJ using two K-wires and suture anchor augmentation of the disrupted CC ligament were included in the study. Patients with Rockwood Type IV and V, and young and active individuals involved in manual labour with type III ACJ dislocation, presenting within three weeks of injury underwent surgical stabilisation. Patients treated elsewhere, chronic ACJ dislocation (>3-week-old injury), patients who had undergone previous surgery on the affected shoulder and those with lateral end of clavicle fractures were excluded from the study. Demographic data and the details regarding the surgical procedure were retrieved from the hospital medical records. Radiographic data were retrieved using Picture Archiving and Communication System (PACS). Pre-operatively, standardised AP view of the clavicle with shoulder joint and AP stress view of bilateral clavicle with patient holding 3kg weight in both hands were performed as a routine in our hospital. Three patients, who were initially classified as Type III injury were re-classified as type V following stress views. All the patients were operated by two trained senior surgeons.

**Fig. 1.**
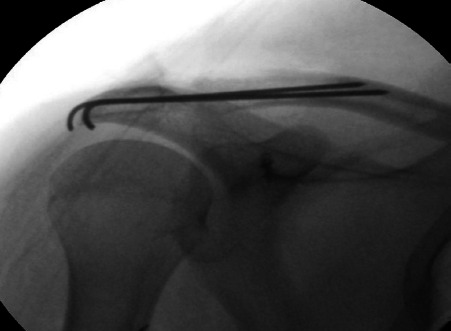
Fluroscopic image showing transfixation of ACJ with K wires and position of suture anchor in the coracoid process.

**Fig. 2.**
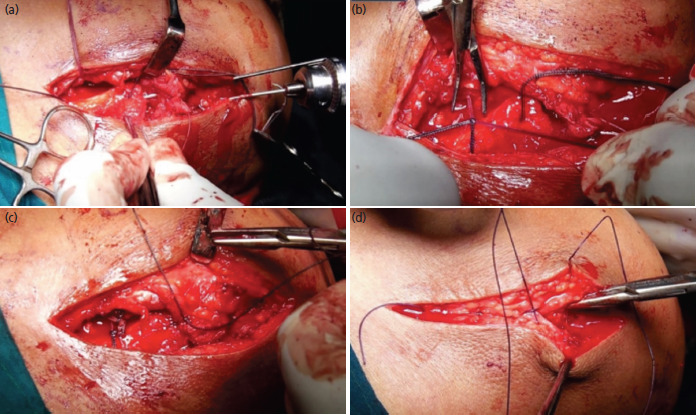
(a) Intra-operative photograph showing transfixation of acromioclavicular joint with two k wires, (b) tying of suture limbs over the clavicle, (c) repair of acromioclavicular joint capsule and (d) closure of deltotrapezial layer.

Modified Roberts or supraclavicular approach^14^ was used for exposure. The 20° beach chair position was used and a 6cm long curvilinear incision was made over ACJ and extended to lateral third of clavicle. Thick flaps were raised. Disruption in the attachment of deltopectoral fascia and insertion of deltoid and trapezius, if any, were noted. Base and elbow of the coracoid process were identified by blunt dissection. Double loaded 5mm single metal suture anchor was placed in the base of coracoid and position confirmed under image intensifier. Two limbs of the sutures were brought around the clavicle from behind and two limbs from the front. Sleeve of trapezius attached on the posterior aspect of lateral end of clavicle was retained if intact. ACJ was reduced under direct vision and stabilised using two K-wires (1.8mm) across the ACJ under image intensifier (Fig. 1). One end of the K-wires was engaged into the cortex of the clavicle to prevent migration and the other end was cut, bent, and buried under the deltoid muscle. All four limbs of the sutures were then tied around the clavicle to maintain the reduction. The ACJ capsule was repaired with absorbable sutures (vicryl 2-0). Deltopectoral fascia and the disrupted delto-trapezial muscles were repaired using the same (Fig. 2). Wound was closed over a drain which was removed the next day.

Passive and active assisted forward flexion and abduction up to 90° was started from the first post-operative day. Arm sling was applied at other times for a total of three weeks. Patients were asked not to carry weight and to avoid overhead abduction for six weeks. Patients were advised to undergo K-wire removal under local anaesthesia at eight weeks of follow-up in the outpatient clinic.

**Fig. 3.**
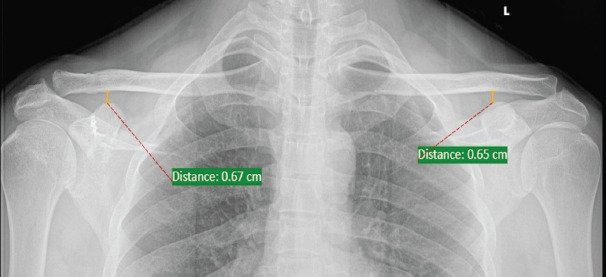
Stress radiograph after two years post-op showing ACJ articulation.

Regular follow-up was done at 2, 8, 12 and 24 weeks, and then on a yearly basis. Standard AP radiograph of the operated clavicle with shoulder joint was taken during each visit. At the end of two years, AP stress radiograph of both clavicle including bilateral shoulder with patient holding 3kg of weight in both hands was repeated to look for any loss of correction (Fig. 3). In the pre-operative, immediate postoperative, and end of two years’ post-operative radiographs, the coracoclavicular distance was measured and side to side comparison was done.

We defined loss of reduction as 100% increase in CC distance compared to the unaffected side in the final follow-up. Measurements were taken by two independent investigators twice at eight weeks apart. Of the 37 patients, 5 were lost for follow-up. Thirty-two cases who had minimum follow-up of 24 months or more were considered for radiological and functional evaluation. Functional evaluation was done using Disabilities of Arm, Shoulder, and Hand Score questionnaire (DASH), Oxford Shoulder Score (OSS) and Constant Shoulder Score (CSS) by a senior Orthopaedic resident.

Descriptive statistics were used to describe demographic data of age, gender, affected side, clinical and radiological outcomes, and functional scoring systems. Inter and intra-observer reliability were analysed by Interclass correlation coefficient. Pearson Correlation coefficient was used to find the correlation between radiological findings and functional scoring. Functional scoring with respect to the Rockwood classification which is an ordinal data was analysed using Kruskal Wallis test. Statistical analysis was done using SPSS *software v 20.0* IBM Corporation. *A p-value* <0.05 was considered as significant.

## Results

Of the 32 patients considered for the study 30 were males and 2 were females. Right upper limb was affected in 17 patients and left in 15 patients. Mean age of the patients was 40±14.02 years. As per the Rockwood classification 9/32 were type III, 11/32 were type IV and 12/32 were type V (Table I). There was a mean delay of 3.96±3.88 days for fixation after injury. K-wires were removed after 3.22±1.15 months. Mean follow-up was 30.59±7.25 months.

**Table I:** Demographic, radiological data, and complications of 32 acromioclavicular dislocation injuries

Variables	Result
No of patients	32
Male : Female	30 : 2
Right : Left	17 : 15
Rockwood classification	
Type III	9
Type IV	11
Type V	12
Coracoclavicular (CC) distance	
Unaffected side	7.55 (±1.93) mm
Affected side	
a) At presentation	13.92 (±4.94) mm
b) Immediate post-op	7.63 (±2.08) mm
c) Latest follow-up	9.36 (±2.75) mm
Complications	
Broken K wires	3
Loss of reduction	1
Ossification of CC ligaments	1

Excellent inter and intra-observer agreement was noted in the radiological measurements. Mean CC distance on the unaffected side was 7.55±1.93mm and on the affected side pre-operatively was 13.92±4.94mm. In the immediate postoperative radiograph, the mean CC distance on the operated side was 7.63±2.08mm. In the final follow-up, the mean CC distance was 9.36±2.75mm. At final follow-up, mean DASH score was 4.67±4.18, Oxford shoulder score was 44.06±2.44 and Constant shoulder score was 86.37±5.81. There was no statistically significant difference in the DASH, CSS, and OSS scores between Rockwood types III, IV and V though type IV injuries had a slightly lower DASH score. There was no significant correlation between final correction achieved and functional scores. Also, there was no correlation with the side of affection and functional scores (Table II).

**Table II:** Functional outcome of 32 patients

	DASH	Oxford Shoulder Score (OSS)	Constant shoulder score (CSS)
Overall outcome	4.67 (±4.18)	44.06 (±2.44)	86.37 (±5.81)
Rockwood type			
III	6.94 (±4.40)	43.77 (±1.78)	84.77 (±7.15)
IV	2.39 (±2.78)	43.45 (±1.57)	87.36 (±5.80)
V	5.05 (±4.30)	44.83 (±3.35)	86.66 (±4.94)
Rockwood type^a^ vs	0.055	0.15	0.61
Final correction achieved^b^ vs	0.228	0.144	0.08
Affected side right or left^c^ vs	0.938	0.565	0.120

* p< 0.05; ^a^ : Kruskal-Wallis test, ^b^ : Pearson Correlation, ^c^ : Student t test, DASH : Disabilities of Arm Shoulder and Hand Questionnaire

In three patients the K-wires had broken without loss of reduction. One among them was reluctant to get it removed even after explaining the complications while the other two got the K-wires removed as a day care procedure in the OT. Another patient was noted to have loss of reduction after K-wire removal. However, he was clinically asymptomatic and did not want to undergo any further intervention. We also observed ossification of the coracoclavicular ligament in a patient.

## Discussion

Though there are an array of treatment options for ACJ dislocations, the highlight of this technique is that both acromioclavicular and coracoclavicular ligament disruptions are addressed with the most cost effective and easily executable techniques individually, making it an ideal technique to be learnt and reproduced for regular practice without speciality training. Furthermore, it showed maintenance of reduction of ACJ after the bifocal fixation, at the end of two years follow-up, with good functional and radiological outcome and negligible complications.

Acromioclavicular joint dislocations account for 8-12% of all dislocations, which is an understatement as many Rockwood type I and II injuries go unreported or unnoticed^2,5^. These injuries are most seen in 2nd to 4th decade and are about 5 to 8 times more common in males than females^10,16^. They mostly happen because of high energy injury during road traffic accidents (RTA) or contact sports. Direct injury due to fall on apex of the shoulder or indirect injuries due to fall on outstretched hand are the two common modes of injury^16^. These injuries are associated with intra-articular glenohumeral pathologies in about 0% to 42.8% of patients^16^. Significance of addressing the glenohumeral pathologies is currently undetermined as these injuries could be incidental findings due to degenerative changes^16^. Clinical evaluation and stress radiographs are the reliable diagnostic modalities.

There is a consensus for treating Rockwood type I and II injuries non-operatively^3,4^. Due to the severity of the injuries, there is also consensus to surgically treat Rockwood type IV and V injuries and so is the case for type III injuries in young active patients, athletes and heavy manual labourers^5,7,17^. Bosworth introduced the screw fixation technique from clavicle to the coracoid which gave good results^18^. Bicortical purchase in the coracoid gives better pull-out strength for this screw. But, due to the rigid nature of the fixation there were many complications including loss of reduction, fracture of the coracoid and clavicle and implant breakage^18^. Subsequently several techniques have been described in the literature to stabilise the joint, each having its own advantages and disadvantages. No technique has been found to be the best to treat this injury^2-19^.

Many techniques using autologous or allogenic tendon grafts, sutures, synthetic ligaments, and cords as a sling around the coracoid and fixed to the clavicle have been described in the literature^20^. Fracture or osteolysis of the coracoid and clavicle, loss of reduction due to stretching of the graft, technical difficulties, infections, allergic reactions, donor site morbidity and potential injury to the neurovascular bundle while passing the sling under the coracoid are some of the described complications of these procedures^6,20^. A recurrence rate of 20-30% is reported within one year after reconstructive procedures^20-22^.

K-wire fixation alone or with sutures either around the K-wires or through bone tunnels in acromion and clavicular hook plate are the options available for the fixation across AC joint^23-26^. Technical simplicity, sleeker implant, absence of the implant after its removal at 8-10 weeks, easy availability and cost effectiveness make K-wire fixation still the widely used option^5^. Breakage and migration of the K-wires, loss of reduction and need of an additional minor procedure to remove the K-wires are the described complications^24,26^. Greater range of movement of shoulder joint, negative intrathoracic pressure, regional bony erosion, and gravity are suggested as reasons for breakage and migration of K-wires^26^. In the early phase of our study, we had 3 cases with wire breakage when we were using 1.5mm K-wires. This prompted us to start using 1.8mm K-wires and subsequently no wire breakage was noted. Usage of hook plate is associated with repeat surgery to remove the implant, osteolysis and fracture of the acromion and impingement of the rotator cuff^11,23,27^. Unlike hook plates, K-wires can be removed in the out-patient clinic under local anaesthesia.

As early anatomical fixation of the ACJ dislocations offer better outcome, non- anatomical procedures like Weaver Dunn procedure, coracoacromial ligament transfer and other reconstruction procedures are reserved for chronic cases and have unpredictable results^5^.

Recently arthroscopic procedures using flip button, graft ropes and suture anchors have been described with good outcome^5,9^. Minimal invasive nature without need of implant removal and ability to address shoulder intra-articular injuries are the cited advantages^16^. Loss of reduction due to the failure of the fixation devices, suture abrasion, fracture of clavicle/coracoid and sinking in of the devices into the clavicle/coracoid due to osteolysis and device pull through are the described complications^7,8,20,26-28^. Disruption of the accessory restraining structures like deltopectoral fascia, trapezius, and deltoid muscles from their insertion on the clavicle and superior AC ligament are the causes for excessive displacement and instability seen in the Rockwood type IV and V injuries^2,13,20^. Repair of these structures is not possible with arthroscopic procedures, and it also has a steep learning curve^5^. In the current study, we take the suture cords around the clavicle. Therefore, no drill holes are made in the clavicle, thereby decreasing the risk of iatrogenic fractures. Also, we leave intact a 1.5cm sleeve of trapezius attached on the posterior aspect of lateral end of clavicle which will prevent inadvertent lateral slippage of the suture cords during overhead movements.

**Fig. 4.**
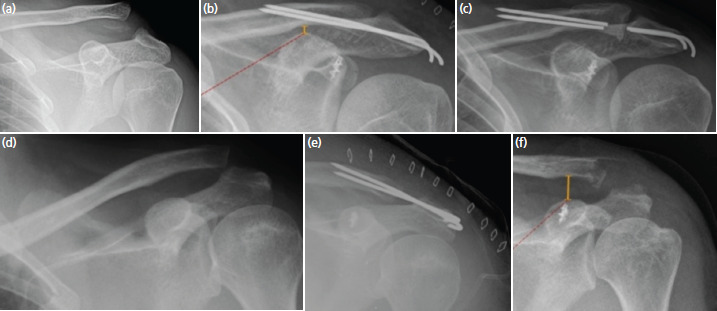
Anteroposterior radiographs of acromioclavicular joints showing post-operative complications –K wire breakage; (a) pre-op, (b) immediate post-op, (c) follow-up. Loss of reduction following K wire removal (d) pre-op, (e) immediate post-op, (f) follow-up.

In our study, we achieved bifocal fixation by using a suture anchor for CC ligament reinforcement, in addition to temporary K-wires for the AC joint stabilisation along with repair of the AC joint stabilising structures. Suture anchor has adequate pull-out strength comparable to native CC ligaments. They are easy to use and avoid potential injury to neurovascular structures while passing sutures under the coracoid^10,20,29^. As per the biomechanical studies, bifocal stabilisation at AC joint and CC region increases the strength of fixation^12,29,30^. Also, acute augmentation, act as a guide for better alignment and healing of the remnant torn ligament fibers along the sutures. We have removed the K-wires at an average of 3.22±1.15 months after the index procedure which is later than the recommended 8 weeks. The main reason for delay in removal of K-wires was non-compliance of the patients for the suggested protocol of K-wire removal. We had three patients with broken K-wires. Thin K-wires, delay in the removal of the K-wire, difficulty in restraining the patients and their non-compliance for the restricted mobilisation and activities involving the shoulder might be the contributing factors. We had three patients who were reluctant to undergo K-wire removal of which one patient had broken K-wires despite explaining the risks involved. Using thicker K-wires (1.8mm or 2mm), getting bicortical purchase across the joint, restriction of heavy overhead activities till removal of K-wires and proper counselling regarding the need of K-wire removal will reduce the incidence of K-wire related complications. If the K-wires are left protruding a bit out of the cortex of clavicle, it is still amenable for removal in the likelihood of its breakage (Fig. 4a-c). Delaying K-wire removal till eight weeks seems to be useful as it facilitates capsuloligamentous healing and its maturation, and hence lessens the risk of loss of reduction. This is evident by loss of reduction in only one patient in our series (Fig. 4d-f).

In our study, neither the severity of injury nor the status at the final follow-up had any effect on the functional evaluation. Our study had a DASH score of 2.39 (±2.78) for type IV injuries which is slightly better than that of type III or type V injuries. This is mainly because of the two outliers in the study, considering it is a subjective scoring, excluding which the DASH score for type IV injuries is 4.12 (±1.05), which is in accordance with the average DASH score in our study. Our findings are consistent with the other studies in the literature which have evaluated different fixation techniques^17-19,27,28^. In other words, our surgical technique if meticulously performed can be employed in all grades of ACJ dislocation (except type 6 which was not included in this study) and helps to achieve good functional stability.

Novelty and strength of our technique lies primarily in the fact that it is economical and can be performed by any Orthopaedic surgeon without formal training in arthroscopy, shoulder surgery or sports medicine. Also, issues like second surgery to remove the implant (hook plate), damage to cuff (hook plate), fracture/osteolysis of clavicle (dog buttons), fracture of acromion (hook plate) are not there. Thirdly, both CC ligamentous stability and ACJ capsular repair is achieved which is not possible with purely arthroscopic technique.

The limitations are pertaining to the retrospective nature of the study. A multicentric randomised controlled trial with larger number of cases would further validate the data. We did not measure the antero-posterior translation in the final follow-up as axillary views were not done. Though there were no cases who presented with pain during the study period post-surgery, the short follow-up precludes the recognition of long-term complications such as ACJ arthritis.

## Conclusion

Treatment for ACJ dislocations should be devised to achieve a stable joint by restoring the function of the acromioclavicular and coracoclavicular ligaments. Bifocal fixation restores the functional stability of the disrupted ACJ. The described surgical technique, if meticulously performed helps to achieve good functional outcome irrespective of the grade of ACJ injury. Adequate radiological reduction, good functional outcome, shorter learning curve and simplicity of execution makes this frugal technique an undemanding one for use in regular practice.
